# Research Progress on the Mechanisms of Protocatechuic Acid in the Treatment of Cognitive Impairment

**DOI:** 10.3390/molecules29194724

**Published:** 2024-10-06

**Authors:** Shuzhi Liang, Zhongmin Zhao, Leilei Liu, Yan Zhang, Xijian Liu

**Affiliations:** 1 College of Traditional Chinese Medicine, Shandong University of Traditional Chinese Medicine, Jinan 250300, China; liang19861401632@163.com (S.L.);; 2 The Youth Research and Innovation Team of TCM for the Prevention and Treatment of Cardiovascular and Cerebrovascular Diseases, Shandong University of Traditional Chinese Medicine, Jinan 250300, China

**Keywords:** cognitive impairment, procatechuic acid, mechanism of action

## Abstract

Cognitive impairment (CI) is a type of mental health disorder that mainly affects cognitive abilities, such as learning, memory, perception, and problem-solving. Currently, in clinical practice, the treatment of cognitive impairment mainly focuses on the application of cholinesterase inhibitors and NMDA receptor antagonists; however, there is no specific and effective drug yet. Procatechuic acid (PCA) possesses various functions, including antibacterial, antiasthmatic, and expectorant effects. In recent years, it has received growing attention in the cognitive domain. Therefore, by summarizing the mechanisms of action of procatechuic acid in the treatment of cognitive impairment in this paper, it is found that procatechuic acid has multiple effects, such as regulating the expression of neuroprotective factors, inhibiting cell apoptosis, promoting the autophagy-lysosome pathway, suppressing oxidative stress damage, inhibiting inflammatory responses, improving synaptic plasticity dysfunction, inhibiting Aβ deposition, reducing APP hydrolysis, enhancing the cholinergic system, and inhibiting the excitotoxicity of neuronal cells. The involved signaling pathways include activating Pi3K-akt-mTor and inhibiting JNK, P38 MAPK, P38-ERK-JNK, SIRT1, and NF-κB/p53, etc. This paper aims to present the latest progress in research on procatechuic acid, including aspects such as its chemical properties, sources, pharmacokinetics, mechanisms for treating neurodegenerative diseases.

## 1. Introduction

Cognitive impairment (CI), a primary mental health disorder that mainly affects cognitive abilities (including learning, memory, perception, and problem-solving), represents the earliest clinically detectable stage of dementia. It is frequently observed in disease complications such as stroke, cerebral infarction, and diabetes. The main manifestations are the progressive weakening of memory and the gradual deterioration of cognitive functions [1]. With the intensification of aging, the prevalence of mild cognitive impairment has been increasing annually. CI patients require lifelong medication and need assistance in daily life, imposing a heavy medical burden on both patients and their families. The pathological characteristics of cognitive impairment can be manifested as atrophy of the medial temporal lobe and hippocampus as well as varying degrees of sclerosis, β-amyloid plaques, neurofibrillary tangles, granule cell degeneration, and apoptosis of hippocampal neurons, etc. [2]. Currently, in clinical treatment, cholinesterase inhibitors such as donepezil are commonly used to inhibit AChE activity, slowing the decomposition of acetylcholine in the synaptic cleft and thereby increasing the content of ACh to improve the cognitive function of patients with Alzheimer’s disease. NMDA receptor antagonists like memantine are employed to regulate the development of neuronal dendrites [3]. However, the former can lead to adverse reactions such as diarrhea, muscle cramps, fatigue, nausea, vomiting, and insomnia, while the latter can cause respiratory depression. Hence, how to effectively prevent and treat CI is a current research focus. Procatechuic acid has multiple functions such as antibacterial, antiasthmatic, and expectorant effects, and has gradually gained attention in the cognitive field in recent years [4].

## 2. Chemical Properties of Protocatechuic Acid

**Figure 1:**
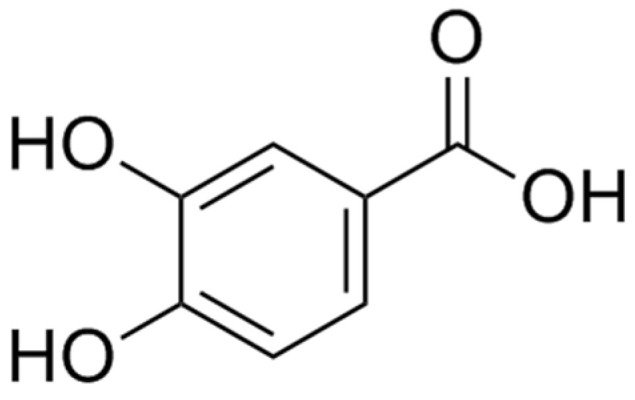
Molecular structure of protocatechuic acid.

Protocatechuic acid (PCA), also known as 3,4-dihydroxybenzoic acid, is a natural phenolic acid with the Molecule ID of MOL000105. It is a white-to-brown crystalline powder that undergoes color change in air. The density is 1.54 g/cm^3^, the molecular formula is (HO)_2_C_6_H_3_COOH, the molecular weight is 154.12, and the melting point is approximately 200 °C. The molecular structure is depicted in Figure 1. It is soluble in ethanol and ether. Currently, research on the functions of PCA mainly focuses on its antioxidant activity, inhibition of tumor cell proliferation [5], and anti-diabetes [6]. In recent years, PCA has been recognized as having neuroprotective and cognitive-enhancing effects [7]. This article reviews the literature on the treatment of cognitive impairment with PCA discovered in the past five years, providing the latest comprehensive evaluation for future clinical research on PCA for neurodegenerative diseases.

## 3. Sources of Protocatechuic Acid

### 3.1. Direct Sources

PCA is present not only in berries and red wine but also in traditional Chinese medicines such as rose [8], wolfberry [9], schizandra [10], pueraria root [11], scutellaria barbata [12], eucommia ulmoides [13], acanthopanax senticosus [14], smilax glabra [15], prunella vulgaris [16], solanum nigrum [17], hedyotis diffusa [18], plantago seed [19], dried tangerine peel [20], euphorbia pekinensis [21], cynomorium songaricum [22], rheum officinale [23], salvia miltiorrhiza [24], phellinus igniarius [25], ephedra [26], sterculia lychnophora [27], and cinnamon [28]. The content of PCA varies in different traditional Chinese medicines, see Table 1. The factors causing the content differences include temperature, pressure, origin of medicinal materials, and the composition and concentration of extraction solvents [8,9,10,11,12,13,14,15,16,17,18,19,20,21,22,23,24,25,26,27,28].

### 3.2. Indirect Sources

**Figure 2:**
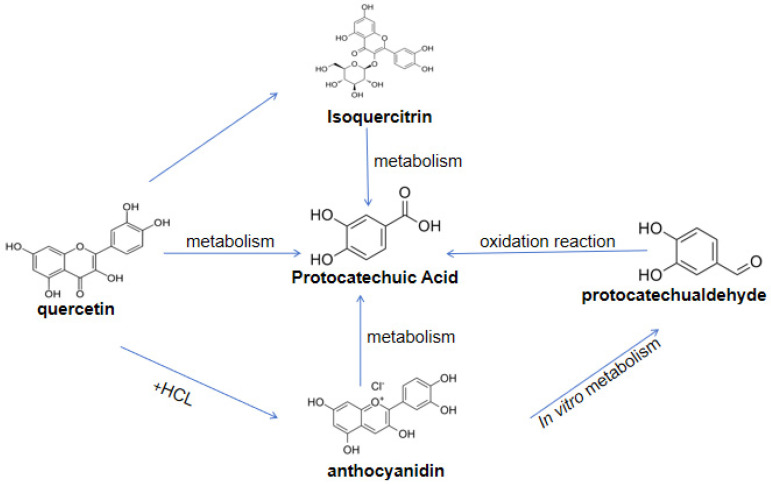
Metabolic diagram of protocatechuic acid.

A small portion of phenolic acids and aldehydes—such as anthocyanin [29], quercetin [30], protocatechuic aldehyde [31], and isoquercitrin [32]—is absorbed through the small intestine, while the majority reaches the colon intact and undergoes intestinal fermentation to be transformed into metabolites such as PCA. Moreover, the transformation conditions are associated with pH value and intestinal flora. It has been confirmed that PCA is transformed in neutral and alkaline fluids and within the intestinal flora. For instance, anthocyanin is stable in acidic environments but its b-ring cleavage occurs under neutral and alkaline pH conditions, decomposing into PCA [29]; therefore, quercetin [30], protocatechuic aldehyde [31], and isoquercitrin [32] can be decomposed by the intestinal microbiota in the human body, and the resulting metabolites include PCA, as depicted in Figure 2.

### 3.3. Pharmacokinetics

**Figure 3:**
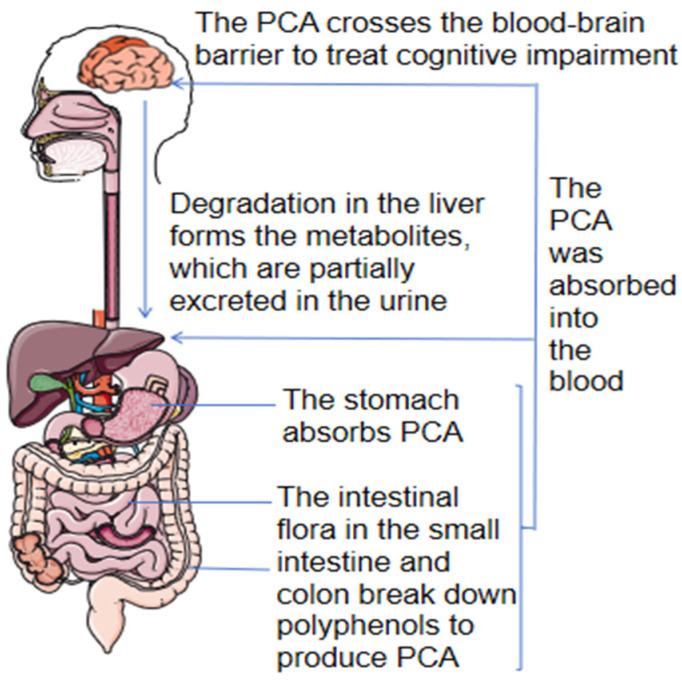
Pharmacokinetics of protocatechuic acid.

Oral PCA is absorbed through the stomach and reaches the liver via the portal vein, where it undergoes metabolism to form active metabolites. The primary metabolic pathway involves the combination of hydroxyl groups on protocatechuic acid with sulfate and glucuronic acid to generate O-methylated conjugates. The methyl metabolite C8H8O6S of PCA was detected in urine, while the methyl metabolites C_8_H_8_O_6_S, C_8_H_8_O_7_S, C_6_H_6_O_5_S, and C_14_H_16_O_10_ of PCA were detected in plasma. No metabolites were detected in the feces of rats [33], suggesting that this component is absorbed and metabolized in the bloodstream of rats and is not excreted through feces. An important basis for PCA in treating cognition is its relatively high permeability, enabling it to pass through the blood–brain barrier via passive diffusion [34]. Oral administration of a 50 mg/kg aqueous solution of PCA can reach the pharmacologically effective concentration of PCA in the body within 5 min and remains stable in human plasma for 24 h. However, it decomposes rapidly and in a dose-dependent manner in mouse plasma [35]. Additionally, PCA demonstrates favorable safety, inhibits apoptotic markers of liver cells, and reduces the extent of liver and kidney damage [36]. There are no studies reporting adverse effects on liver and kidney functions. The pharmacokinetics are depicted in Figure 3.

## 4. The Mechanisms by Which Protocatechuic Acid Treats Cognitive Impairment

**Figure 4:**
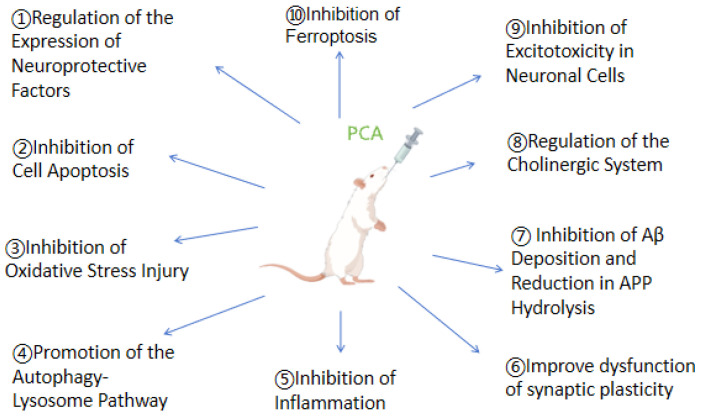
Ten mechanisms of protocatechuic acid in treating cognitive impairment.

Figure 4 displays the various mechanisms involved.

### 4.1. Regulation of the Expression of Neuroprotective Factors

It has been discovered in research that PCA can regulate the expression of neuroprotective factors to enhance cognition. Krzysztoforska, K. [37] experimentally demonstrated that in the brains of rats administered with PCA, the level of DA increased and the level of AGE decreased. PCA improves memory ability by reducing the level of AGE, decreasing Tet2, activating NF-κB and NLRP3, and enhancing intestinal permeability [38]. As an inhibitor of monoamine oxidase (MAO) and dopamine β-hydroxylase, PCA effectively elevates dopamine (DA) levels by obstructing the conversion of DA into norepinephrine (NA) via dopamine β-hydroxylase and preventing further oxidative deamination by MAO. This mechanism subsequently leads to the upregulation of downstream receptor gene expression for Drd1 and Drd4, mediated by PCA, thereby playing a crucial regulatory role in cognitive decline.

Krzysztoforska, K. [39] further discovered that PCA led to an elevation in histidine concentration within both the hippocampus and prefrontal cortex. This increase in histidine levels ameliorated mitochondrial damage induced by the synergistic effects of diabetic hypoglycemia, facilitating mitochondrial energy utilization for the comprehensive maintenance of calcium (Ca^2+^) homeostasis, neuronal growth, and axonal branching, thereby ensuring effective synaptic transmission [40]. This phenomenon is associated with the buffering and antioxidant properties of histidine; notably, histidine treatment significantly upregulates histamine receptors (H1R and H2R) following combined exposure to diabetic hypoglycemia—receptors that play crucial physiological roles in memory consolidation, sleep–wake regulation, appetite control, and stress response within the brain [41].

Song, Y. [42] demonstrated that PCA treatment enhances the expression of BDNF/pro-BDNF in the hippocampus and prefrontal cortex of ischemia-reperfusion rats. This intervention not only modulates the activity of BACE1 in these brain regions but also elevates ADAM10 activity, as well as increases phosphorylation levels of Akt at Ser473 in the hippocampus and GSK3β at Ser9 in both the hippocampus and prefrontal cortex. BDNF is known to regulate GSK3β signaling pathways, thereby contributing to cognitive function improvement [43].

Krzysztoforska, K. [44] reported that PCA administration significantly elevated 5-HT (5-HIAA/5-HT) turnover within the hypothalamus and prefrontal cortex. The activation of 5-HT1AR appears to reduce Iba1, GFAP, and TNF-α expression, indicating its role in inhibiting microglial and astrocytic activation induced by aβ1-42 [45].

Kale, S. [46] found that PCA administration markedly increased CREB expression in cortical tissues subjected to ischemia-reperfusion injury. Elevated CREB levels were associated with reduced ROS production and inhibited apoptosis among primary neurons in mice. Notably, CREB knockout downregulated components of the PGA-CAMKIV signaling pathway while decreasing PKA, CaMKIV, CREB, pCREB, arc, and c-fos protein expressions within primary neurons—ultimately impairing memory functions [47]. Thus, enhancing CREB expression can facilitate improvements in learning and memory capabilities.

In conclusion, PCA ameliorates cognitive dysfunction through elevations in DA, histidine, BDNF, 5-HT, and CREB levels while concurrently reducing AGE.

### 4.2. Inhibition of Cell Apoptosis

Yin, X. [48] discovered that PCA could increase the expression level of Bax and decrease the expression level of Bcl-2 by acting on the Bcl-2 family proteins in neuronal cells. The integrity of the mitochondrial membrane was altered, resulting in an increased release of ROS and cytochrome c, activating caspase-9 and caspase-3, thereby activating the mitochondrial apoptotic pathway. Additionally, the reduction in ROS induced by PCA could inhibit the activation of caspase-3, promote the proliferation of neural stem cells, and prevent the apoptosis of neural stem cells [49].

Kho, A.R. [50] employed the TSQ fluorescence method to reveal that PCA could lower the intracellular zinc level in the CA1 region of the hippocampus. It inhibits the mitochondrial energy processes, including the dissipation of the mitochondrial membrane potential (MMP), leading to ATP depletion and consequent neuronal death [51].

Liu, Y.M. [52] found that PCA could prevent the collapse of the mitochondrial membrane potential induced by ichthyotoxin and inhibit the opening of the mitochondrial permeability transition pore (PTP), thereby causing apoptotic substances such as cytochrome c to be released from the mitochondria into the cytoplasm, and subsequently inactivate caspase-3-like enzymes.

**Figure 5:**
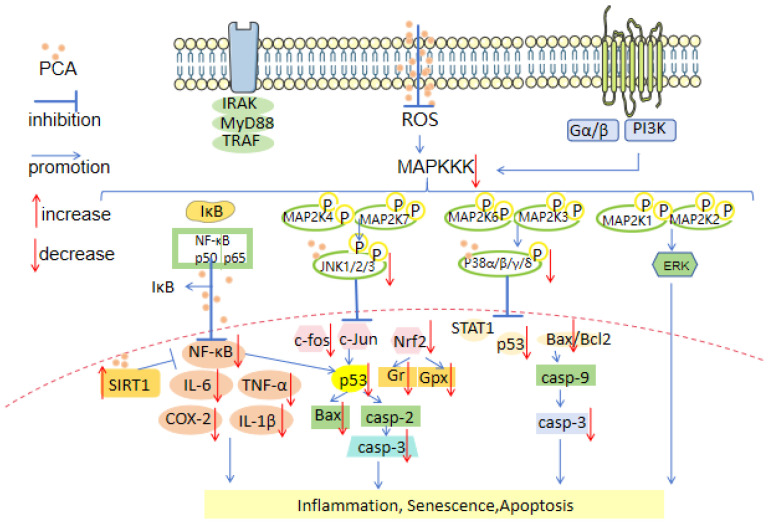
The P38-ERK-JNK and NF-κB pathways.

The mitogen-activated protein kinase (MAPK) superfamily comprises three members: the classical MAPK (also referred to as ERK), JNK, and p38 [53]. Yin, X. [54] discovered that PCA mainly treats cognitive disorders by inhibiting the JNK and P38 MAPK signal transduction pathways. PCA inhibits the apoptosis of hippocampal and prefrontal cortex neurons by reducing the phosphorylation of JNK/P38 and downstream targets c-fos, Bax, and cleaved caspase-3 in the hippocampus and prefrontal cortex. The pathway is depicted in Figure 5.

### 4.3. Inhibition of Oxidative Stress Injury

Due to the brain’s high oxygen utilization rate, high non-heme iron content that promotes the generation of reactive oxygen species (ROS), and high polyunsaturated fatty acids content, the brain is at high risk of oxidative damage [55]. PCA possesses dose-dependent antioxidant capacity. Compared with standard antioxidants, PCA exhibits more effective antioxidant potential in both lipid and aqueous media [56]. Protocatechuic acid is an effective phenolic acid antioxidant. Its antioxidant activity mainly stems from its phenolic hydroxyl group and the activation of endogenous antioxidant enzymes. It can inhibit lipid peroxidation and has moderate hydrogen peroxide and DPPH radical scavenging activity in vitro [57].

PCA treatment has been shown to attenuate the increase in xanthine oxidase (XOD) activity [54], thereby inhibiting XOD-catalyzed purine oxidation and the production of superoxide radicals as by-products.

In oxidative reactions, PCA is capable of inhibiting the overexpression of inducible nitric oxide synthase (iNOS) to generate a large amount of NO, thereby suppressing the reaction of NO with superoxide radicals (O^2−^) to form the peroxynitrite anion (ONOO^−^), inhibiting the oxidative reaction process and alleviating neuronal cell damage [58].

PCA elevates superoxide dismutase (SOD) [59] to catalyze the disproportionation of superoxide radicals (O^2−^) into hydrogen peroxide. Additionally, PCA can raise glutathione peroxidase (GSH-PX) [59] and catalase (CAT) [59], further converting hydrogen peroxide into water, thereby eliminating superoxide radicals within the body [60]. This decreases the oxidation of unsaturated fatty acids into lipid peroxides and subsequently inhibits the further decomposition of these peroxides into malondialdehyde (MDA), thereby reducing the reactions of malondialdehyde (MDA) with phospholipids and proteins to form neurotoxic lipofuscin [61], improving learning and memory.

PCA is able to lower hydrogen peroxide [62] levels and inhibit its reaction with intracellular reduced iron ions through the Fenton reaction to generate highly toxic hydroxyl radicals, suppressing toxicity to neuronal cells in brain tissue. Since neuronal membranes contain a large amount of oxidation substrates such as polyunsaturated fatty acids and have low catalase activity, they are more susceptible to oxidative damage than other parts [63].

Adeyanju, A.A. [64] discovered that PCA downregulates neuronal NF-κB expression. The activation of NF-κB functionally disrupts or inhibits p53 activity, thereby leading to a reduction in cisplatin-induced neurodegenerative damage. PCA exerts its antioxidant activity in the brain through NF-κB/P53 pathways. The pathway is shown in Figure 5.

### 4.4. Promotion of the Autophagy-Lysosome Pathway

Cellular autophagy refers to the process by which cells use lysosomes to degrade and recycle damaged organelles and macromolecular substances within the cell. Abnormalities in the autophagy-lysosome pathway, such as the deposition of Aβ1-42, the accumulation of p62 protein, and the hyperphosphorylation and aggregation of Tau, can all result in cognitive dysfunction. When the autophagy process of hippocampal neurons is abnormal, the homeostasis of neuronal cells is disrupted and the cognitive function of the body is affected. The presence of LAMP-1 enables autophagosomes to fuse with lysosomes, introducing the encapsulated cellular components into lysosomes for degradation [65].

P62 is a protein closely related to the autophagy process. As a linker protein, it helps combine the labeled intracellular waste with autophagosomes and promotes their degradation and clearance. When autophagy is activated, P62 is degraded in autolysosomes and can be continuously consumed as autophagy progresses [66], and its expression level indirectly reflects the level of autophagosome clearance.

The mammalian protein microtubule-associated protein 1 light chain 3 (LC3) can specifically bind to the autophagosome membrane and is located on the surface of pre-autophagosomes and autophagosomes, participating in the regeneration and extension of the autophagosome membrane. There are two convertible forms of LC3-I and LC3-II: When autophagy occurs, LC3-I processed by ubiquitination-like modification binds to phosphatidylethanolamine on the surface of the autophagosome membrane, thereby converting to LC3-II. They are involved in the formation of the autophagosome membrane, and this process marks the occurrence of autophagy [67].

Beclin 1 is a key protein in the autophagy process, which participates in the formation of autophagosomes and the regulation of autophagy [68].

Li, H. [69] demonstrated through immunofluorescence that PCA increased the fluorescence intensities of LAMP1 and lysosomes, enabling autophagosomes to fuse with lysosomes and introducing the encapsulated deposits of Aβ1-42, accumulated p62 protein, and hyperphosphorylated aggregates of Tau into lysosomes for degradation. WB showed an increase in the autophagy-related marker LC3-II/I, a significant decrease in the p62 level, and a significant increase in the levels of LAMP1 and CD proteins, indicating an increase in autophagy levels, a reduction in the deposition of Aβ1-42, the accumulation of p62 protein, and the hyperphosphorylated aggregates of Tau, thereby improving cognition.

**Figure 6:**
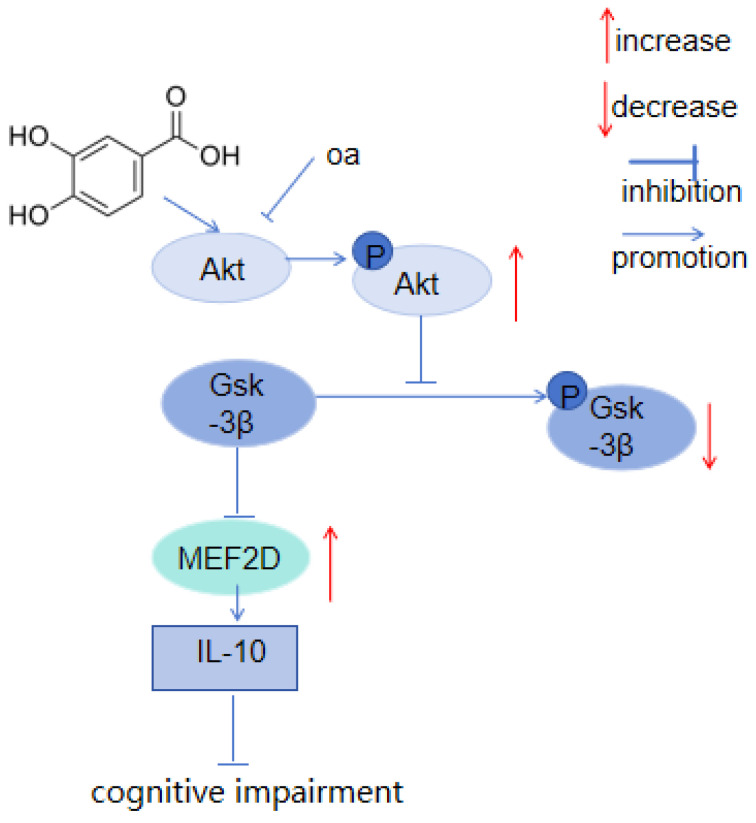
The akt/GSK-3β/MeF2d signaling pathway.

Huang, L. [70] discovered that PCA significantly reduced the expression of Beclin-1, activated the akt/GSK-3β/MeF2d signaling pathway, activated Akt through inducing phosphorylation, and that the phosphorylation of Akt promoted the phosphorylation of GSK-3β, thereby reducing the inhibitory effect of GSK-3β on MEF2D. The increase in the nuclear concentration of MEF2D increased the expression of IL-10 and decreased the expressions of IL-1β, IL-6, and TNF-α. It induces protective autophagy to treat cognitive dysfunction. The pathway is shown in Figure 6.

### 4.5. Inhibition of Inflammation

Neuroinflammation refers to a series of immune responses mediated by neuroimmune cells within the central nervous system. Neuroimmune cells, including microglia and astrocytes, activate inflammatory mediators that can compromise the integrity of the blood–brain barrier and white matter in the brain, ultimately leading to cognitive impairment [71]. Amyloid-beta (Aβ) has been shown to activate pro-inflammatory pathways involving cyclophilin A and matrix metalloproteinase 9 (MMP-9) in pericytes, exacerbating inflammatory damage [72].

Xi, Z. [48] demonstrated that PCA downregulates both P38-ERK-JNK and NF-κB signaling pathways in treating cognitive impairment. PCA treatment inhibited phosphorylated MAPK production in a dose-dependent manner, prevented NF-κB translocation from the cytoplasm to the nucleus, and suppressed the expression levels of IL-1β, IL-6, and TNF-α.

Kaewmool, C. [73] found that PCA inhibited the release of IL-1β, IL-6, TNF-α, iNOS, and COX-2 in BV2 microglia through modulation of the SIRT1/NF-κB pathway; it increased SIRT1 levels while reducing p65 acetylation. This resulted in decreased neuroinflammation as well as protection for both the blood–brain barrier and white matter—ultimately improving cognitive function.

### 4.6. Improve Dysfunction of Synaptic Plasticity

Dendritic spines are the postsynaptic sites of excitatory synapses and an essential component of neuronal synaptic plasticity. As the physiological basis for the formation of cognitive functions, neural synaptic plasticity participates in the pathological process of cognitive impairment formation [74]. Lee, S.H. [75] discovered that the MAP2 immunoreactivity (IR) in the hippocampus and cortex of rats with traumatic brain injury treated with PCA significantly increased, suggesting that PCA inhibits the loss of dendrites. Guan, S. [76] experimentally demonstrated that the dendritic spine-like structures in the elongated neurites of differentiated cells in the PCA group were more prominent, indicating that PCA influences the terminal differentiation stage of neurons, induces neuronal maturation, and effectively promotes neurite growth.

Synaptophysin (SYP) is located on the synaptic vesicles of the presynaptic membrane. By binding to the lipid of the synaptic vesicle membrane, it promotes the movement of synaptic vesicles to the presynaptic membrane and the release of neurotransmitters— while also participating in the occurrence and differentiation of synapses—and is one of the indicators showing changes in synaptic plasticity [77]. Glial Fibrillary Acidic Protein (GFAP) is the support system or “scaffold” for cells and nuclei in astrocytes. One of its functions is to provide mechanical support to the plasma membrane that contacts other cells or the extracellular matrix. An increase in GFAP production can cause hypertrophy, proliferation, and migration of astrocytes, forming reactive astrogliosis [78]. Excessive gliosis promotes reactive glial cell proliferation-induced glial scarring, which acts as a mechanical barrier to hinder the regeneration of myelin and axons, affecting the structural repair and functional recovery of neural tissue [79]. Yin, X. [54] utilized immunohistochemistry and found that PCA elevated the SYP in neuronal cells of the hippocampus and prefrontal cortex of rats, promoting the movement of synaptic vesicles to the presynaptic membrane and the release of neurotransmitters, thereby facilitating the occurrence and differentiation of synapses; further, it reduced the expression of GFAP in the cortex and hippocampus, inhibited the formation of reactive astrogliosis, suppressed the generation of glial scars, promoted the regeneration of myelin and axons, and facilitated structural repair and functional recovery. Additionally, PCA can also promote the growth of dendritic spine-like structures in elongated neurites, reduce dendritic loss, be beneficial for neuronal synaptic plasticity, and thereby improve learning and memory.

### 4.7. Inhibition of Aβ Deposition and Reduction in APP Hydrolysis

Aβ is generated by proteolytic processing of amyloid precursor protein (APP) through β-secretase, β-site APP cleaving enzyme 1 (BACE1), and γ-secretase. Excessive extracellular Aβ levels in the brain can inhibit long-term potentiation (LTP) in the hippocampus and affect memory function [80]. Moreover, it leads to Aβ aggregation, forming soluble Aβ oligomers with high neurotoxicity that can further reaggregate into fibrils to form amyloid plaques [81]. The aggregation and accumulation of Aβ drive tau phosphorylation promote the formation of neurofibrillary tangles, and subsequently induce synaptic toxicity, neurotoxicity, and ultimately cognitive impairment [82]. Rummel, N.G. [83] discovered that small, highly hydrophobic Aβ oligomers cause lipid peroxidation, resulting in the production of 4-hydroxynonenal (HNE) that leads to oxidative modification and dysfunction of membrane lipids and cytosolic resident proteins. Oxidatively modified proteins can cause neuronal death as well as thinning of the hippocampus and frontal cortex, thereby resulting in cognitive impairment. Song, Y. [42] found that PCA could attenuate Aβ deposition and APP in the hippocampus and cerebral cortex of AβPP/PS1 mice. Huang, L. [70] demonstrated that the levels of p-tau, Aβ_42_, and β-secretase in the PCA treatment group decreased in a dose-dependent manner. Hornedo-Ortega, R. [84] observed using transmission electron microscopy that the number of Aβ_1-42_ aggregates decreased with increasing PCA concentration, and the monomeric form of α-synuclein increased.

In conclusion, PCA is capable of attenuating APP, reducing β-secretase, thereby reducing APP hydrolysis, lowering extracellular Aβ levels in the brain, inhibiting the formation of soluble Aβ oligomers with high neurotoxicity, suppressing tau phosphorylation, inhibiting the formation of neurofibrillary tangles and, subsequently, inhibiting synaptic and neurotoxicity, which is conducive to learning and memory.

### 4.8. Regulation of the Cholinergic System

Acetylcholine (ACh) is a crucial neurotransmitter in the central cholinergic system, capable of transmitting neuronal information and contributing to enhanced learning and memory abilities. The impairment of cholinergic neurons in the brain can result in cognitive disorders [85]. Under the action of choline acetyltransferase (ChAT), the acetyl group on acetyl coenzyme A is transferred to choline to generate acetylcholine. In the synaptic cleft, acetylcholine is degraded by acetylcholinesterase (AChE) and the activity changes in both enzymes jointly maintain the dynamic equilibrium of acetylcholine in brain tissue [86]. Acetylcholine can increase the activity of α-secretase, thereby facilitating the processing of APP along the non-amyloidogenic generation pathway and reducing the formation of Aβ [87].

Consequently, the PCA treatment group, by activating choline acetyltransferase [88] and significantly reducing the activity of acetylcholinesterase, elevates the level of acetylcholine in the synaptic cleft, improves normal neural transmission in rats [89], reduces the formation of Aβ and, subsequently, ameliorates cognitive impairments in vivo.

### 4.9. Inhibition of Excitotoxicity in Neuronal Cells

Glutamate exerts its physiological effects by binding and activating ligand-gated ion channels [ionotropic glutamate receptors (iGluRs)] and a class of G protein-coupled receptors [metabotropic glutamate receptors (mGluRs)]. Timely clearance of glutamate from the synaptic cleft is necessary. High levels of extracellular glutamate overactivate glutamate receptors, leading to excitotoxicity in the central nervous system. Additionally, the increase in extracellular glutamate concentration inhibits cystine uptake through the cystine/glutamate antiporter (system Xc-), resulting in glutathione depletion and glutamate oxidative toxicity [90]. NMDARs are ionotropic receptors with the highest affinity for glutamate and are double-gated channels with high calcium conductance. Excessive activation of NMDARs by glutamate leads to a large influx of Ca^2+^, activating various degradation processes—such as endonucleases involved in the necrotic process—and causing mitochondrial swelling and cytochrome c release from mitochondria, thereby initiating the apoptotic process that will lead to thinning of the hippocampus and frontal cortex brain regions and results in cognitive impairment [91]. Ban, J.Y. [92] found that PCA can inhibit the increase in Ca^2+^ concentration and glutamate induced by Aβ (25–35).

Therefore, PCA promotes astrocytes to recycle glutamate from the synaptic cleft through glutamate reuptake and then converts it to glutamine via glutamine synthetase, reducing the level of glutamate in the synaptic cleft and inhibiting glutamate receptors. On the one hand, it reduces the excitotoxicity of neuronal cells, and on the other hand, it inhibits the influx of Ca^2+^, thereby inhibiting various degradation processes, preventing mitochondrial swelling, inhibiting the release of cytochrome c from mitochondria—thus preventing the initiation of the apoptotic process, protecting the hippocampus and frontal cortex, and improving cognitive ability.

The glutamate and GABAergic systems are representative excitatory and inhibitory nervous systems, and their interaction and maintenance of a balanced state have significant implications for maintaining learning and memory functions. The weakened inhibitory function of GABAergic neurons can cause excessive excitation of excitatory neurons, thereby disrupting the excitation–inhibition balance state, generating excitotoxicity, ultimately triggering abnormal activities at the neural network level and leading to cognitive dysfunction [93]. Mert, H. [94] experimentally demonstrated that PCA can increase the GABA level in the hippocampus.

Therefore, PCA can increase the GABA level in the hippocampus, enhance the inhibitory excitatory function, and weaken excitotoxicity. Adefegha [95] found that PCA can significantly increase the activity of Na^+^/K^+^ ATPase, thereby improving the dysfunction of the Na-K pump under conditions of brain tissue ischemia and hypoxia, inhibiting the disorder of Na^+^ and K^+^ concentrations inside and outside cells, inducing the generation of various enzymes and ATP, preventing the activation of voltage-sensitive Ca^2+^ channels, preventing a large influx of Ca^2+^, reducing the release of neurotransmitters such as glutamate, and reducing the cytotoxic effects on neuronal cells, which is beneficial for cognitive dysfunction [96].

In summary, PCA can significantly increase the activity of Na^+^/K^+^ ATPase, thereby improving the dysfunction of the Na-K pump under conditions of brain tissue ischemia and hypoxia, inhibiting the disorder of Na^+^ and K^+^ concentrations inside and outside cells, inducing the generation of various enzymes and ATP, preventing the activation of voltage-sensitive Ca^2+^ channels, preventing a large influx of Ca^2+^, reducing the release of neurotransmitters such as glutamate, and reducing the cytotoxic effects on neuronal cells. At the same time, PCA promotes astrocytes to recycle glutamate from the synaptic cleft through glutamate reuptake and then converts it to glutamine via glutamine synthetase, reducing the level of glutamate in the synaptic cleft and inhibiting glutamate receptors, reducing the excitotoxicity of neuronal cells. PCA can also increase the GABA level in the hippocampus, enhance the inhibitory excitatory function, and weaken excitotoxicity. Through the above three mechanisms, PCA weakens the cytotoxic effects on neuronal cells and improves cognitive dysfunction.

### 4.10. Inhibition of Ferroptosis

Ferroptosis is an iron-dependent novel form of programmed cell death, distinct from apoptosis and autophagy, and is associated with various diseases such as degenerative disorders and stroke. The main mechanism of ferroptosis is that, under the action of divalent iron or lipoxygenase, the highly expressed unsaturated fatty acids on the cell membrane are catalyzed, resulting in lipid peroxidation and thereby inducing cell death. Additionally, it is characterized by glutathione depletion, a reduction in the core regulatory enzyme GPX4 of the antioxidant system (glutathione system), and accompanied by elevated levels of reactive oxygen species and malondialdehyde. Muley MM et al. discovered that PCA induces the phosphorylation of the transcription factor Nrf2 mediated by JNK. Along with the intense activation of the transcription factor, a large amount of Nrf2 translocates to the nucleus, increasing the level of active phosphorylated JNK and enhancing glutathione reductase (GR) [97], glutathione peroxidase (GPX) [59], and the ferroptosis factor glutathione (GSH) [50] in neural stem cells, while reducing lipid hydroperoxide (LPO) [62] and the ferroptosis factor malonic dialdehyde (MDA) [98] in neural stem cells. GPX4, as an antioxidant defense enzyme, is regarded as a key regulatory factor for inhibiting ferroptosis and can eliminate excessive toxic lipid hydroperoxides, thereby improving oxidative stress damage in cells; however, this process requires the participation of glutathione as a cofactor [99]. Glutathione, as the substrate of GPX4, participates in the intracellular antioxidant system. Direct or indirect inhibition of glutathione biosynthesis can decrease GPX4 activity and promote the occurrence of ferroptosis [100]. Moreover, GSH is also a natural ligand for Fe^2+^ in the labile iron pool (LIP) of neurons. The binding of GSH to Fe^2+^ in LIP prevents iron oxidation, which not only maintains the solubility of Fe^2+^ but also prevents Fe^2+^ from generating hydroxyl radicals as a catalyst. The inhibition of GSH synthesis directly or indirectly reduces the antioxidant capacity of cells [101]. Nrf2 is a key target for alleviating cell damage and also a crucial regulatory factor for ferroptosis [102]. Studies have shown that nuclear factor erythroid 2-related factor 2 (Nrf2) can promote the expression of GSH, GR, and GPX4, increasing the antioxidant capacity of cells. On the other hand, Nrf2 can also inhibit the occurrence of ferroptosis by promoting the transport of intracellular iron ions and reducing the accumulation of intracellular iron ions [103].

Therefore, the mechanism by which PCA inhibits ferroptosis is as follows: PCA induces the phosphorylation of the transcription factor Nrf2 mediated by JNK. Along with the intense activation of the transcription factor, a large amount of Nrf2 translocates to the nucleus, increasing the level of active phosphorylated JNK and promoting the biosynthesis of GSH. On the one hand, it increases the binding of GSH to Fe^2+^ in LIP to prevent iron oxidation, inhibits the catalysis of highly expressed unsaturated fatty acids on the cell membrane under the action of divalent iron or lipoxygenase, and suppresses lipid peroxidation, thereby protecting neuronal cells; on the other hand, it promotes the synthesis of GPX4, converting the peroxide bonds of lipid peroxidation into hydroxyl groups, eliminating excessive toxic lipid hydroperoxides, and maintaining the homeostasis of the membrane lipid bilayer, thereby protecting neuronal cells and improving cognitive dysfunction.

## 5. Summary

With the growth of the elderly population and the extension of life expectancy, the issue of age-related neurodegenerative processes will spread and exert social and economic influences. Hence, seeking new therapeutic approaches has become an indispensable task for modern medicine.

This article, by summarizing the mechanism of action of PCA in cognitive impairment, discovers that PCA can function through multiple pathways such as regulating the expression of neuroprotective factors, inhibiting apoptosis, promoting the autophagy-lysosome pathway, suppressing oxidative stress damage, inhibiting inflammatory responses, ameliorating synaptic plasticity dysfunction, inhibiting Aβ deposition and reducing APP hydrolysis, improving the cholinergic system, suppressing excitotoxicity of neuronal cells, and inhibiting ferroptosis. The action pathways involve activating Pi3K-akt-mTor and inhibiting JNK, P38 MAPK, P38-ERK-JNK, SIRT1, and NF-κB/p53, etc. The action pathways of PCA are extensive, encompassing multiple pathways and targets. However, as the current research on PCA is in its infancy, there are relatively few clinical and basic studies on the application of PCA in the treatment of patients with cognitive impairment. Research on the safety, toxicity, teratogenicity, and carcinogenicity of PCA is lacking. Moreover, the research mechanisms for inhibiting the effects of certain pathways are still needed, and further experiments and clinical studies are required to fully determine the therapeutic efficacy of PCA.

## Figures and Tables

**Table 1:** Protocatechuic acid content in different traditional Chinese medicines.

Name	Content (mg/g)	Detection Methods	References
rose	0.476–1.632	Extraction using an ethanol aqueous solution at 180 °C	[8]
wolfberry	0.1819–0.9114	HPLC-MS	[9]
schizandra	0.0722–0.3569	HPLC-DAD	[10]
pueraria root	0.04	HPLC-/MS	[11]
scutellaria barbata	0.0361–0.066178	Supercritical CO2 Extraction	[12]
eucommia ulmoides	0–0.20694	HPLC	[13]
acanthopanax senticosus	0.003165–0.02312	HPLC-DAD	[14]
smilax glabra	66	HSCCC	[15]
prunella vulgaris	27	HPLC-DAD-ESI	[16]
solanum nigrum	4.1–48	LC-MS	[17]
hedyotis diffusa	0.561	UHPLC-MS/MS	[18]
plantago seed	0.001881–0.004856	QAMS	[19]
dried tangerine peel	0.043–0.075	GC-MS, UPLC	[20]
euphorbia pekinensis	0.02603–0.361	LC-MS/MS	[21]
cynomorium songaricum	0.012134–0.025287	LC-MS/MS	[22]
rheum officinale	0.03899	HPLC	[23]
salvia miltiorrhiza	0.915	cce-UV	[24]
phellinus igniarius	0.016–0.032	UHPLC-QqQ	[25]
ephedra	0.05–0.75	HPLC-DAD	[26]
sterculia lychnophora	0.0219	UPLC-MS/MS	[27]
cinnamon	0.1931–0.2278	LC-HRMS	[28]

## Data Availability

The data presented in this study are included in the article.

## Abbreviation List

**Abbreviation**	**Full title**	**Abbreviation**	**Full title**
CI	Cognitive impairment	PCA	Protocatechuic acid
AGEs	Advanced glycation end products	DA	Dopamine
5-HT	5-hydroxytryptamine	BDNF	Brain-derived neurotrophic factor
CREB	cAMP-response element binding protein	NF-κB	Nuclear factor κB
PKA	Protein kinase A system	XOD	Xanthine oxidase
LC3	Microtubule-associated protein 1 light chain 3	MMP-9	Matrix metalloproteinase 9
HNE	4-hydroxynonenal	ChAT	Choline acetyltransferase
AChE	Acetylcholinesterase	GR	Glutathione reductase
GPX	Glutathione peroxidase	GSH	Glutathione
LPO	Lipid hydroperoxide	MDA	Malonic dialdehyde
LIP	Labile iron pool		
